# Evaluation of pretreatment methods for filamentous fungal detection

**DOI:** 10.1038/s41598-024-61517-1

**Published:** 2024-05-13

**Authors:** Xiaoli Jiang, Daiwen Xiao, Hua Yu, Anhua Sun, Qin Liu, Tao Liu

**Affiliations:** 1 Department of Experimental Medicine, The People’s Hospital of Jianyang City, Chengdu, 641400 China; 2 Department of Laboratory Medicine and Sichuan Provincial Key Laboratory for Human Disease Gene Study, Sichuan Provincial People’s Hospital, University of Electronic Science and Technology of China, Chengdu, 610072 China

**Keywords:** Filamentous fungi, MALDI-TOF MS, Formic acid sandwich method, Dispersion method, Extraction method, Biological techniques, Microbiology

## Abstract

In order to obtain the best mass spectrometry identification results for using the most appropriate methods in clinical practice, we explore the optimal pretreatment methods for different species and morphologies of filamentous fungi. 98 fungal strains were treated with formic acid sandwich method, dispersion method, extraction method, and other methods using a medium element mass spectrometer (EXS3000) as a platform. Each strain had three targets, and the identification rates and confidence differences under different pre-treatment methods were compared to evaluate the identification effects of these methods. The mass spectrometry identification rates of 98 filamentous fungi obtained after pre-treatment with formic acid sandwich method, dispersion method, and extraction method were 85.71%, 82.65%, and 75.51%, respectively. The identification rate of the formic acid sandwich method was significantly higher than the other two methods (P < 0 005) has the best identification ability and the obtained confidence is also higher than the other two methods. The use of formic acid sandwich method for mass spectrometry identification of filamentous fungi can achieve ideal identification results, which is suitable for mass spectrometry identification of filamentous fungi in conventional laboratories.

## Introduction

In recent years, with the increasing use of broad-spectrum antibiotics and the use of anti-tumor drugs, immunosuppressive drugs, etc., the incidence of invasive filamentous fungal infections in clinical practice has been increasing year by year. Invasive filamentous fungal infections have complex clinical manifestations, difficult diagnosis and treatment, and are characterized by high infection rate, high misdiagnosis rate, and high mortality rate. There are many types of filamentous fungi, and the drug resistance spectrum of different strains within the same genus may vary. Rapid and accurate identification of filamentous fungi can guide clinical rational selection of antifungal drugs^1–3^. Traditional morphological identification requires fungal isolation and culture, which is less accurate and time-consuming, and is prone to identification errors or unidentification^4^. In addition, although the G(1-3-β-D glucan) test, GM(galactomannan) test or Aspergillus antigen antibody test in serological testing do not require fungal isolation and culture, and can quickly indicate the possibility of fungal infection, they are susceptible to a variety of factors and present false positive and false negative. In addition, there is cross-reaction in fungal serological tests, which makes it difficult to determine the species of fungi^5,6^ and cannot provide accurate etiological diagnosis for clinical practice, so antifungal drugs cannot be used reasonably. Although molecular sequencing is the "gold standard", its operation is cumbersome and expensive, the identification time is long, and it requires high laboratory conditions and the technical level of operators, which is not suitable for routine microbiology laboratory operations. In recent years, matrix assisted laser desorption ionization time of flight mass spectrometry (MALDI-TOF MS) has become a revolutionary technology for microbial identification, starting to be used for clinical identification of filamentous fungi. It has the advantages of low cost, accurate and fast identification, and good repeatability. However, compared to bacteria, mass spectrometry identification of filamentous fungi has always been a difficult point in clinical identification. Filamentous fungi have thick cell walls, difficult to break, and difficult to extract proteins, which affects the quality of mass spectrometry protein profiles and leads to poor identification results^7^. The above is the main reason why the mass spectrometry identification of filamentous fungi is not yet widely used in microbiology laboratories. There are no standardized guidelines for mass spectrometry pretreatment methods for filamentous fungi, and seeking efficient pretreatment methods is the key to the experiment. The essence of obtaining a spectrum for MALDI-TOF mass spectrometry identification is mainly to obtain the distribution of ribosomal proteins in the strain. The biggest factor affecting its accuracy is the release of bacterial proteins at the target site. Switching between different pre-treatment methods is to obtain the most complete protein types and appropriate protein concentrations of the strain. Due to the different morphologies of different genera of filamentous fungi, the difficulty of obtaining proteins varies greatly when identifying filamentous fungi. Therefore, MALDI-TOF mass spectrometry may have certain differences in identifying different genera of filamentous fungi. In order to seek the optimal pre-treatment method for filamentous fungi, this study will use the Zhongyuan Mass Spectrometer (EXS3000) as a platform to analyze and compare the differences in the identification results of different species of filamentous fungi using three pre-treatment methods: formic acid sandwich method, dispersion method, and extraction method. The aim is to explore the optimal pre-treatment method for filamentous fungi and provide timely pathogenic evidence for clinical practice.

## Materials and methods

98 fungal strains were treated using methods such as formic acid sandwich, dispersion, and extraction, with three targets per strain. The accuracy and scores of different types of morphology were compared to evaluate the identification effectiveness of these methods.

Filamentous fungi need to choose an appropriate growth time (generally 3–7 days, for some dark fungi for 10–15 days) to ensure that the fungal body is fresh. When some fungal strains were extracted, the dispersion of fungal bodies was not enough and magnetic beads could be added for grinding extraction.

### Source of culture

The comparison strains that have been genetically sequenced provided by Sichuan Provincial People’s Hospital have 98 samples, covering several common clinical filamentous fungi such as *Aspergillus, Mucor, Penicillium, Fusarium, Cladosporium, Trichosporum and Microsporum bacteria*.

### Reagents and instruments

Zybio mass spectrometer (Zhongyuan Huiji Company, model EXS3000), light microscope (Olympus Japan, model: BX53), mold incubator (Shanghai Boxun Industrial Instrument Co., Ltd., model: HPX-9272MBE), sterile pre-assembled extraction tubes (Zhongyuan Huiji Company), disposable target plates (Zhongyuan Huiji Company), matrix solution (Zhongyuan Huiji Company, batch number 230401), Escherichia coli ATCC 25922 (Zhongyuan Huiji Company), fungal sample lysate Ι(70% formic acid,Zhongyuan Huiji Company, batch number 230201), fungal sample lysate II (acetonitrile , Zhongyuan Huiji Company, batch number 5206001), mold extraction reagent I (Zhongyuan Huiji company, batch number 2220901), mold extraction reagent II (Zhongyuan Huiji company, batch number 2220901), sabouraud dextrose agar medium (Sabouraud dextrose agar, SDA; Jiangmen Kailin Company, batch number 230224).

### Strain culture and identification

Transfer the strain to SDA and incubate it in a mold incubator at 28 ℃ for 2 days. Scotch tape^8^ was used to observe the morphological features of hyphae, spores, and spore production structures under a light microscope for identification. For some strains with poor sporulation, the small culture method^9^ was used for further identification.

### MALDI-TOF MS pretreatment method

#### Formic acid sandwich method

1. Add 1 μL of formic acid solution dropwise to the target.
2. Pick a small amount of fungal bodies were taken from the tip of a 10μL straw.
3. Spread the fungal body on the target in a clockwise direction.
4. Cover with 1 μL of formic acid after drying.
5. Cover with 1 μL of matrix solution after drying.
6. After drying, it will be tested on the machine.

#### Dispersion method

1. Add 200 μL of mold extraction reagent I and 200 μL of mold extraction test II to the mold extraction tube.
2. Immerse the sampled swab in the mixed extraction reagent.
3. Scrape the freshly cultured fungi from the SDA plate with a sampling swab and rinse them into the extraction solution, repeated several times until the solution is cloudy
4. Vortex and shake to mix well, no need to centrifuge, add 1 μL of suspension solution dropwise to the sample target, and add 1 μL of matrix solution dropwise to cover the sample after drying.

#### Extraction method

1. Pick 1–5 mg of fresh fungi in 1.5 mL centrifuge tube (avoid picking the medium), add 300 μL ultra-pure water, and fully suspend.
2. Add 900 μL of absolute ethanol and mix well.
3. Centrifuge at 12,000 rpm for 2–3 min, discard the supernatant and centrifuge for 1 min, remove the residual supernatant, and dry the precipitate at room temperature for 5 min (ethanol volatilization can dry).
4. Add 20 μL of fungal sample lysate Ι, mix well with pipette gun and leave at room temperature for 5 min.
5. Add 20 μL of fungal sample lysate II, mix thoroughly, and centrifuge at 12,000 rpm for 2 min. Pipette 1 μL of the supernatant to the sample target, cover 1 μL of matrix solution after drying, and test it on the machine after drying.

#### Extraction method (magnetic bead grinding) – as a supplement to 1.4.3

1. Pick 1–5 mg of fresh fungi in 1.5 mL centrifuge tube (avoid picking the medium), add 300 μL ultra-pure water, and fully suspend.
2. Add 900 μL of absolute ethanol and mix well.
3. Centrifuge at 12,000 rpm for 2–3 min, discard the supernatant and centrifuge for 1 min to remove the residual supernatant. Dry and precipitate for 5 min at room temperature (ethanol can be volatilized and dried).
4. Add 20 μL of fungal sample lysate Ι and 20 μL of fungal sample lysate II.
5. Add an appropriate amount of glass strain, use a homogenizer to shake and crush for 3 min, and let stand for 5 min.
6. A After thorough mixing, centrifuge at 12,000 rpm for 2 min; Absorb 1 μ L supernatant to sample target, dry and cover with 1 μ L matrix solution, dried and tested on the machine.

### Statistical analysis

SPSS 21.0 software was used for statistical analysis. This study is counting data, the rate is described, and the chi-square test is used for comparison between groups. The test level α = 0.05.

### Ethics statement

This paper is the original research results independently obtained by all the authors, no manuscripts have been submitted, and has not been published in any form in any language at home and abroad. The content of the paper does not infringe on the copyright and other rights of others. In case of multiple submissions, infringement and other problems, all authors of the paper will bear full responsibility.

## Results

### Identification of different pretreatment methods

According to the data, the pre-treatment method using formic acid sandwich method has a good identification effect on filamentous fungi, with an identification rate of 85.71%, followed by the dispersion method with an identification rate of 82.65%. The pre-treatment method using extraction method has a relatively poor identification effect, with an identification rate of 75.51%. See Table 1.

**Table 1:** Identification of different pretreatment methods.

Pre-treatment method	Numbers accurately identified to the species level	Accurately identify the number of genera	Unidentified	Identification rate (%)
Formic acid sandwich method	66	18	14	85.71
Dispersion method	67	14	17	82.65
Extraction method	52	22	24	75.51

### Identification of pretreatment methods for filamentous fungi of different species

According to the results, we can see the pretreatment method with the best identification effect of Aspergillus and Penicillium was formic acid sandwich method, and its identification rates were 94.74% and 83.33%, respectively. The pretreatment method with the best identification effect of Cladospora was the dispersion method, and its identification rate was 88.24%. The identification effect of the three pretreatment methods of Fusarium was good, and the identification rate was 100.00%. The identification results of the three pretreatment methods of Mucormyces and Alternaria were consistent, and the identification rate was 100.00%. See Table 2.

**Table 2:** Identification of pretreatment methods for filamentous fungi of different species.

Genus	Number of samples	Formic acid sandwich method				Dispersion method				Extraction method			
		Green	Yellow	Red	Identification rate (%)	Green	Yellow	Red	Identification rate (%)	Green	Yellow	Red	Identification rate (%)
Aspergillus	38	27	9	2	94.74	26	5	7	81.58	17	12	9	76.32
Fusarium spp	10	10	0	0	100.00	8	2	0	100.00	9	1	0	100.00
Penicillium	12	5	5	2	83.33	8	2	2	83.33	6	2	4	66.67
Cladosporum spp	17	11	1	5	70.59	10	5	2	88.24	8	5	4	76.47
Mucor	3	3	0	0	100.00	3	0	0	100.00	2	1	0	100.00
Alternaria	3	3	0	0	100.00	2	1	0	100.00	2	1	0	100.00
Bottle mold	4	4	0	0	100.00	4	0	0	100.00	3	0	1	75.00
other	11	5	1	5	54.55	5	1	5	54.55	6	0	5	54.55
subtotal	98	68	16	14	85.71	66	15	17	82.65	53	22	23	76.53

## Discuss

Mass spectrometry over flight (MALDI-TOF MS) is a technique for the identification of pathogenic microorganisms at the proteomic level, which has many advantages such as speed, efficiency, and economy. The most difficult thing to identify filamentous fungi by MALDI-TOF MS technique is the pretreatment to extract proteins, which is the main factor affecting the identification results of filamentous fungi. The results of different filamentous fungal protein extraction methods on the identification of mass spectrometry were quite different. Different bacterial protein extraction protocols directly affect the accuracy of identification^10,11^. The cell wall of filamentous fungi contains more chitin components, and the wall is thick and tough, so it needs to be pretreated to fully rupture the cell wall, and the cytoribosomal protein release can be used for subsequent mass spectrometry analysis. Our study showed that the pretreatment method of formic acid sandwich method had a good identification rate of 85.71% for filamentous fungi, followed by the dispersion method with an identification rate of 82.65%, and the pretreatment method of extraction method had a relatively poor identification effect of 75.51%. The results of the pretreatment methods for different species of filamentous fungi in this study showed that the formic acid sandwich method was the formic acid sandwich method with the best identification effect of Aspergillus and Penicillium, respectively, and the dispersion method was the dispersion method with the best identification effect of *C. cladospora,* and the identification rate was 88.24%. The identification effect of the three pretreatment methods of Fusarium was good, and the identification rate was 100.00%. The identification results of the three pretreatment methods of *Mucormyces* and *Alternaria* were consistent, and the identification rate was 100.00%. This may be less than that of Mucor and Alternaria, which cannot reflect the difference between the three pretreatment methods.. Our study found that the efficiency of protein precipitation was different among different species of filamentous fungi due to the differences in morphology and difficulty of wall breaking, and there were some differences in the identification effect of different pretreatment methods.

Mass spectrometry identification has the highest accuracy, greatly reducing the time required for identification. It can provide timely pathogen diagnosis for clinical practice, help clinical patients choose antifungal drugs correctly, timely control patient infections, and reduce mortality rates. Therefore, considering all factors, it is ideal to culture filamentous fungi in SDA medium for 3 days and extract proteins for mass spectrometry identification using the pre-treatment method of formic acid sandwich.

The limitation of this study is that some filamentous fungi have a relatively low quantity and variety. In future research, we will continue to collect and retain strains, enrich the quantity and variety of filamentous fungi, and include them in the study. Therefore, in subsequent experiments, it is necessary to continue exploring and optimizing various influencing factors for the identification of filamentous fungi by mass spectrometry, in order to better improve the identification rate of filamentous fungi by mass spectrometry. Gradually achieving operational standardization in the identification of filamentous fungi in clinical laboratories^12,13^. It is believed that in the future, MALDI-TOF MS technology will be more and more widely used in the identification of filamentous fungi, and will gradually replace the traditional morphological identification methods.

### Supplementary Information

Supplementary Information.

## Data Availability

All data generated or analysed during this study are included in this published article [and its Supplementary Information files].
